# The critical node detection problem in hypergraphs using weighted node degree centrality

**DOI:** 10.7717/peerj-cs.1351

**Published:** 2023-05-03

**Authors:** Tamás-Zsolt Képes

**Affiliations:** Computer Science, Babes-Bolyai University of Cluj-Napoca, Cluj-Napoca, Romania

**Keywords:** Node detection, Hypergraphs, Centrality measures, Genetic algorithm, Critical nodes, Graph theory, Network analysis

## Abstract

Network analysis is an indispensable part of today’s academic field. Among the different types of networks, the more complex hypergraphs can provide an excellent challenge and new angles for analysis. This study proposes a variant of the critical node detection problem for hypergraphs using weighted node degree centrality as a form of importance metric. An analysis is done on both generated synthetic networks and real-world derived data on the topic of United States House and Senate committees, using a newly designed algorithm. The numerical results show that the combination of the critical node detection on hypergraphs with the weighted node degree centrality provides promising results and the topic is worth exploring further.

## Introduction

Network analysis is an important topic that has received large amounts of attention in recent years, while its applications span multiple fields of study such as telecommunication in Dzaferagic et al. (2018), biology in Albert (2005), ecology in Bascompte (2007), etc.

One of the main problems in network analysis is the Critical Node Detection Problem (CNDP) as described in Lewis & Yannakakis (1980), or in the survey by Lalou, Tahraoui & Kheddouci (2018). In general, the CNDP can be described as the problem of finding a node or a set of nodes, that are considered important or critical, according to a specific metric. The CNDP has had many applications during the past few years such as social network analysis in Borgatti (2006) and Fan & Pardalos (2010), network vulnerability studies in Dinh & Thai (2011) and network risk management in Arulselvan et al. (2007). Furthermore, a survey on the topic of critical element detection is presented in Walteros & Pardalos (2012), where the authors describe, among other critical element types, critical nodes.

The Critical Node Detection Problem has been mostly studied on traditional graphs, directed or undirected, weighted or unweighted as in Shen & Smith (2012), Shen, Smith & Goli (2012) and Marx (2006), *etc*. and the problem has been further optimized in papers like Walteros et al. (2019), where the authors focus on generalizing and optimizing the approach for both CNDP and a more generalized critical structure detection, using a mathematical programming approach. CNDP has also been approached with a memetic search algorithm in Zhou, Hao & Glover (2019), with a specific application for the cardinality-constrained CNP variant. An improvement for the memetic algorithm is presented in Zhou et al. (2021). In Salemi & Buchanan (2022) a distance-based variant to the CNDP is solved which enables an increase in the size of networks that can be reasonably investigated. Also in Zhou et al. (2022) a fast tri-individual memetic search is proposed for the distance-based variant of the CNDP.

Much less focus was given to the CNDP’s application on hypergraphs. A hypergraph can be considered a generalization of a graph, where each edge can connect any number of nodes, and as such, it can provide an interesting application for the CNDP, because the basic definition of a critical node is changed by the hypergraph environment.

While the CNDP has been investigated on hypergraphs in the past, this study differentiates itself from any previous works done on this topic by introducing a more complex metric into the CNDP algorithm with the use of Weighted Node Degree Centrality from Kapoor, Sharma & Srivastava (2013).

The article will proceed with a more in-depth introduction of the proposed problem of CNDP in hypergraphs, including mathematical definitions and improvements on and distinctions from previous work done on the topic. Following this, an introduction to the weighted node degree centrality metric is given and possible applications for this research are discussed. Next, an algorithm will be presented. Finally, numerical results are presented for this approach on a number of synthetic networks and also a case study will be given on US congressional committee networks, followed by conclusions and possible future work.

## Critical Node Detection in Hypergraphs

Firstly, a formal definition must be given to traditional graphs.

Definition 1 *A graph is represented as a G* = (*V*, *E*)* pair, where V is the set of nodes or vertices, while E is the set of edges, which in traditional graph theory are node pairings.*

Hypergraphs described in Berge (1984) can be considered as a generalization of a graph, where each edge can connect any number of nodes. Hypergraphs have been used for example in artificial intelligence Feng et al. (2019), image classification Yu, Tao & Wang (2012), biology Franzese et al. (2019), *etc.*

Definition 2 *A hypergraph is a* $\mathcal{H}=(X,\mathcal{D})$* double, where X* = {*x*_1_, *x*_2_, …, *x*_*n*_}* is the set of nodes,* $\mathcal{D}=&lcub; {D}_{1},{D}_{2},&hellip; ,{D}_{m}&rcub; $* is a set of the subsets of X, denoting the set of hyperedges, n and m refer to the number of nodes and hyperedges respectively.*

**Figure 1:**
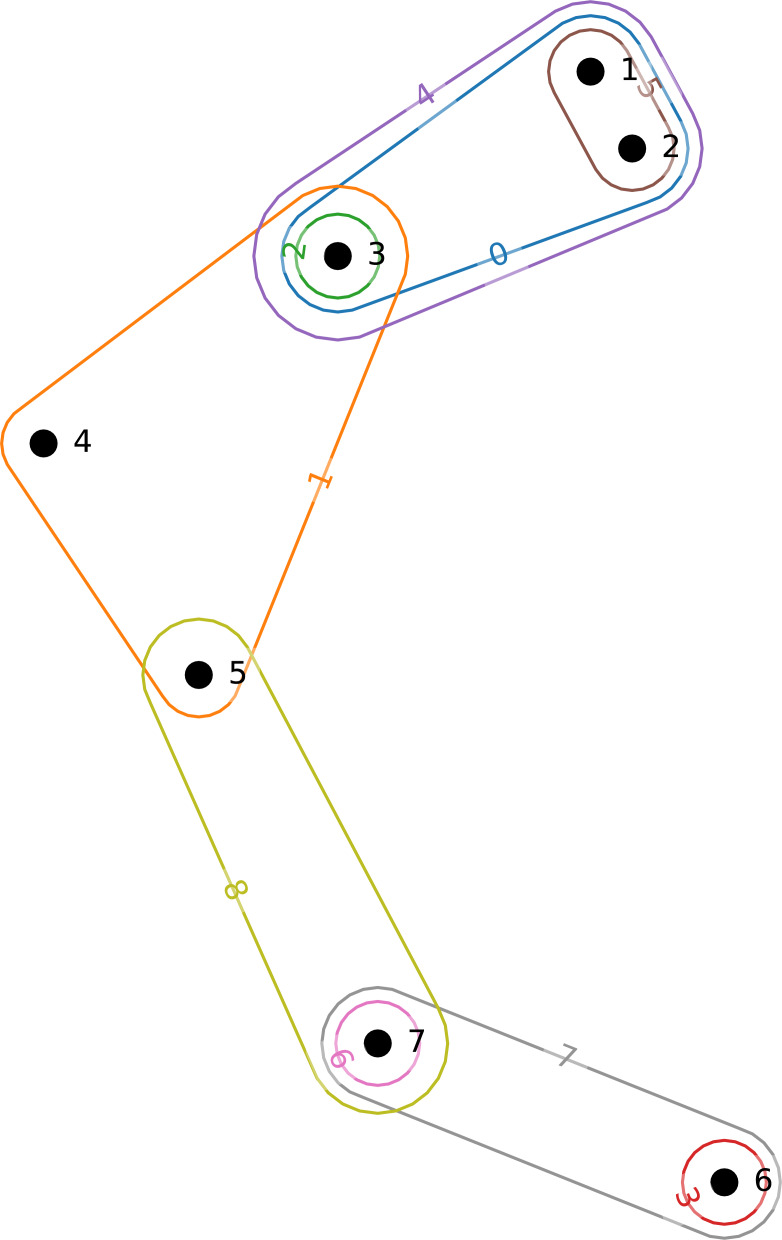
Small example hypergraph.

An example hypergraph is given in Fig. 1. In this example, we can see nine hyperedges labeled from 0 to 8 connecting seven nodes labeled from 1 to 7.

Definition 3 *Having a graph G*(*V*, *E*)*, the Critical Node Detection Problem (CNDP) can be defined as the problem of finding a set of nodes S with the size of k, that when deleted, will maximally degrade G according to a given metric σ*(*G*)*.*

In traditional cases, *σ*(*G*) can be anything from the minimization of a connectivity measure, such as pairwise connectivity, the minimization of the largest connected component, or the maximization of the number of connected components, *etc.*

Previous research already tackled a more simple combination of the CNDP and hypergraphs in Gaskó et al. (2022), and this paper aims to improve and expand the topics researched there.

In Gaskó et al. (2022) the hypergraph representation was not an optimal one. There, a translation was proposed from a hypergraph into a connected web of complete sub-graphs, by replacing every hyperedge with a complete sub-graph, where every node that would participate in an edge, will participate in this sub-graph and will be connected with every other node in the same situation. In the case when a node would participate in multiple hyperedges, the given node will participate in multiple sub-graphs. In this research, a more dedicated method for hypergraph representation is used in the form of the Python package named HyperNetX, developed by the Battelle Memorial Institute, where the hypergraphs are represented by a dictionary-style approach. This approach increases the possibilities of methods that can be applied to a hypergraph, allowing for a more complex *σ*(*G*) to be discussed.

Secondly, a completely new, redesigned, and rewritten algorithm is proposed, the new variant will be discussed in an upcoming section.

Finally, the most significant difference was in the definition of *σ*(*G*). While previously the maximization of the number of connected components in a hypergraph, after the removal of the set *S* of nodes was used as the metric, the current approach to *σ*(*G*) is significantly different and is the main focus of this paper. *σ*(*G*) works by introducing the weighted node degree centrality measure for the CNDP. This new measure is described in the next section.

### Weighted node degree centrality

The weighted node degree centrality was proposed in Kapoor, Sharma & Srivastava (2013), as a natural extension of traditional centrality measures to hypergraphs. The following section will provide a short summary of the proposed metric and how it applies to our study.

Definition 4 * Simple degree centrality for nodes in a hypergraph can be defined as the number of nodes adjacent to the primary node.*

Definition 4 disregards the strength of the ties between nodes, so a weighted degree centrality is proposed to mitigate this problem.

Definition 5 * Weighted node degree centrality can be defined using:*

\begin{eqnarray*}{C}_{d}^{h}(i)={\mathop{\sum }\nolimits }_{j=1}^{N}{\mathop{\sum }\nolimits }_{k=1}^{L}{w}_{k},if\{ {v}_{i},{v}_{j}\} \subset {e}_{k} \end{eqnarray*}

* where, N is the number of nodes, L is the number of hyperedges, e*_*k*_* is a specific hyperedge, w*_*k*_* is the weight of the specific hyperedge and v*_*i*_* is a specific node.*

Multiple hyperedge weights can be described, all of them make use of the frequency of the hyperedge’s appearance or its multiplicity (*m*_*j*_) and the cardinality of the hyperedge (*c*_*j*_), the importance of multiplicity and cardinality can be demonstrated in example 1. The following weights are used in the application:

• **Constant:** *w*_*j*_ = 1
• **Frequency based:** *w*_*j*_ = *m*_*j*_
• **Newman’s definition of ties strength in collaboration networks:** ${w}_{j}= \frac{{m}_{j}}{{|}{c}_{j}{|}-1} $
• **Network theory:** ${w}_{j}=1-(1- \frac{1}{{c}_{j}-1} )^{{m}_{j}}$

Example 1 * Let’s suppose that there are two groups of people D*_1_* and D*_2_.*D*_1_* has 3 members while D*_2_* has 15. The people in D*_1_* meet twice a week while those in D*_2_* meet only once a month. By this allegory, the people in D*_1_* are in a much closer relationship because of two factors, there are fewer of them in the group, so they are supposedly much closer, secondly, they meet more frequently, meaning that their relationship is closer in that regard too. From the hypergraph perspective, D*_1_* and D*_2_* are the hyperedges, the number of people is the cardinality while the number of meetings is the multiplicity.*

In any one instance of the algorithm, a singular weighting scheme is used at a time, incorporating itself into the fitness function. The goal of the fitness calculation is the minimization of the average weighted node degree centrality in the network, after the removal of a set of nodes. A more formal definition of the problem is given in Definition 6.

Definition 6 * Having a hypergraph* $\mathcal{H}=&lcub; X,\mathcal{D}&rcub; $*, where X* = {*x*_1_, *x*_2_, …, *x*_*n*_}* is the set of nodes,* $\mathcal{D}=&lcub; {D}_{1},{D}_{2},&hellip; ,{D}_{m}&rcub; $* is the set of hyperedges, our goal is to minimize the average weighted node degree centrality* ${C}_{d}^{h}(i)$* in the modified network, where i* ∈ *X*∖*S, S being the set of potentially critical nodes.*

## Algorithm

A basic genetic algorithm (GA) is proposed, in order to perform the selection of *k* number of critical nodes from the total node lists. The following details need to be clarified in order to fully understand the created algorithm and the tough process behind it.

### Encoding

The created population for the GA contains a fixed number *pop*_*size* of individuals. These individuals in turn contain *k* number of nodes that are “proposed” as a possible critical node list. Each node is represented by its integer index in the node dictionary, in turn, meaning, that nodes represented with labels can also be processed.

### Fitness

The fitness value for each individual is calculated using the average weighted node degree centrality in the hypergraph $\mathcal{H}$ after the removal of the selected individuals’ nodes from the total node dictionary. This removal modifies the structure of $\mathcal{H}$, in order to not lose information, the fitness calculation is done on a copy of the original hypergraph. Multiple weight calculations are considered, and all of them are described in detail at the weighted node degree centrality explanation.

### Crossover

The crossover process is described in Algorithm 2. It samples a subset of the total population with size *tournament*_*size*, we then sort the selected subset and use the two best individuals, according to their fitness, and we initiate the crossover process using them. The crossover is done by zipping the two individuals’ node lists and then separating them, we attribute special attention to the correctness of the results, *i.e.,* nodes can not repeat in a correct node list. The two new individuals are appended to the child list and the process is repeated *tournament*_*count* times. After crossover completion, the child population is pre-evaluated, in order to skip the evaluation at later steps of the algorithm.

### Mutation

The mutation operator simply selects an individual from the child population and replaces one of its nodes with a new node, taking into account the correctness criteria stated at the crossover operator. The newly mutated individual is then inserted back into the child population. The new individual is also pre-evaluated.

### Selection

The algorithm combines the original population and any newly-created child population, including any possible mutations, after which we trim this new set to the original *pop*_*size*_ using elitism (keeping the best individuals), this will become the new population for the next iteration of the algorithm (a (*μ* + *λ*) selection scheme is used). The selection process also involves an evaluation step at the end, in case any individual needs re-evaluation.

## Experiments and Results

The proposed algorithm depends on a large number of parameters, both in terms of the basic genetic algorithm parameters but also base values for the more unique sections and for the fitness calculations. The following paragraphs will outline the main parameters and the methods of parameter selection for each individual value.

### Population size

The population size parameter refers to the number of individuals considered in each generation of the genetic algorithm. Generally, a larger value provides better results, but having too large of a value inquires a negative trade-off of performance while also providing diminishing returns. Population size also factors into the number of tournament rounds used in the creation of the new population in each generation, meaning that having an excessively large population provides an even bigger penalty in speed. The population size parameter has been chosen as 50 from the pool of 20, 50, and 100 after a round of parameter-setting runs on a few synthetic networks, that have shown no significant difference in results with the 50 and 100 cases, so we opted for the smaller one, to decrease step times.

### Mutation chance

The mutation chance or *p*_*mut*_ gives a percentage chance of a mutation occurring during a genetic algorithm generation. Each generation can have at most one mutation occurring and if a mutation is required then the child selected for mutation and the node replaced by the mutation is selected at random, without any bias, from the children created through the crossover tournament and the nodes that are not present in the selected child respectively. The impact that *p*_*mut*_ has on the algorithm is twofold. Firstly a high mutation chance has an easier time escaping local optimums and moving our search along, resulting in fewer required generations. Secondly, a higher chance of mutation provides volatile results. *p*_*mut*_ was also chosen from a pool of values: 1%, 2% or 5% as a result of the same parameter selection process. The value chosen was 5% because it did the best out of all tested values.

### Generation count

This parameter refers to the number of generations of the GA that we process. This value started out as high as 10,000 but after a few test runs we identified that no more than 200 generations are necessary in most cases that we have studied, so this number was chosen as a stable point and it was not subject to the parameter selection process.

### Probability of selection into the crossover process

The probability of selection into the crossover process or *p*_*cross*_ for short factors into the number of tournament rounds and the number of parents selected to participate in the tournament. This probability should be high, but not 100% in order to exclude some parents from participating and in turn ensure higher variability. This number was also chosen from a pool of possibilities: 60% or 80%. Testing has shown a marginal improvement for the 80% case.

### Tournament size

A round of the crossover tournament always combines two separate individuals to create two new children, but the process of selecting the two participating parents is not trivial. We need to sample the two best parents from those that were selected to participate in the current round, the number of participating parents in any round is the number given by the tournament size parameter. This number was also chosen from a pool of possibilities: 2, 3, or 4. Observations after several parameter-setting runs show, that anything above a value of 2 will provide very similar results. Finally, a value of 3 was chosen.

### Type of weighted centrality

The algorithm also receives as a parameter, the desired type of weighted centrality from the supported list presented in the description of the weighted centrality measure. For the parameter-setting scenario of the GA, a simple constant weight is used to eliminate any variation that this measure would introduce.

### Networks

Some baseline synthetic networks were constructed using LFR benchmarks (https://www.santofortunato.net/resources). These graphs, described in Table 1, were smaller in scale and were mainly used as both proof of concept about the correctness of the algorithm with the different weight types and as a benchmarking tool, for the parameter-setting part of the research process.

In addition to synthetic networks, two real-world networks were used as proof of concept, the data sets were used in Chodrow, Veldt & Benson (2021) and were derived from Congressional data compiled by Charles Stewart and Jonathan Woon. In these networks, nodes represent either members of the US House of Representatives in the first network or members of the US Senate in the second one; while hyperedges correspond to committee memberships. Some basic information about these networks is presented in Table 2.

### Results

**Figure 2:**
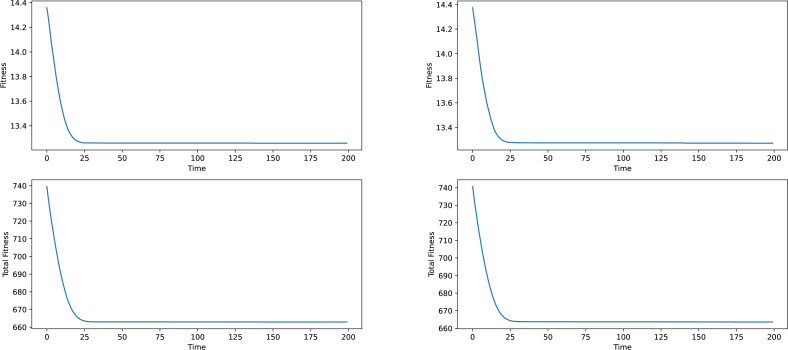
Best individual and total population fitness values over time aggregated for several similar networks, constant and frequency based weights.

**Figure 3:**
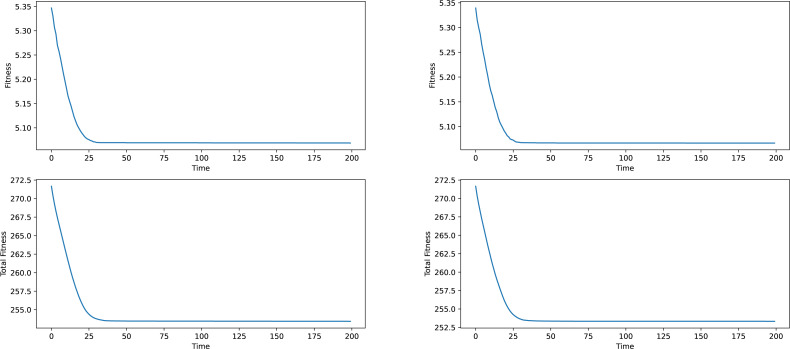
Best individual and total population fitness values over time aggregated for several similar networks, Newman and network-based weights.

Firstly some results on the synthetic networks will be given. The results will not be analyzed in terms of meaning, given the fact, that the data is artificially generated and does not correspond to anything real-world related. The results provided in Figs. 2 and 3 are averages of ten runs, for six similarly generated community networks.

Regarding the results presented in Figs. 2 and 3, we see that the total fitness value of the population and the fitness value of the best individual in the population simultaneously and suddenly lower to a point, typically reached at the 25–50 generation mark, after which a stagnation period is entered. The latest changes happened at around generation 150, which is why a generation number of 200 was chosen. In a local conclusion, it can be said, that the algorithm works in terms of lowering the desired fitness value, indifferent of the used weight type or the network analyzed, which means that this algorithm as a tool of analysis can be useful and can provide interesting results.

#### Comparison between heuristic and GA

Validating the usefulness of the results obtained by the proposed genetic algorithm should be a priority. Since the approach of critical node detection on hypergraphs is a fairly new field of study, no real benchmarks are set. Nevertheless, a comparison of some kind should be considered. This paper presents a comparison between a proposed heuristic algorithm and our GA.

**Table 1:** Synthetic networks and basic properties.

Hypergarph	|*X*|	|*D*|	rank	mean edge size	median edge size
lfr_150_m01_on70_om2	150	150	7	3.86	4
lfr_150_m02_on70_om2	150	150	6	3.69	3
lfr_150_m03_on70_om2	150	150	6	3.76	3
lfr_150_m01_on50_om2	150	150	7	4.00	4
lfr_150_m02_on50_om2	150	150	6	4.04	4
lfr_150_m03_on50_om2	150	150	6	3.90	4

**Table 2:** Real networks and basic properties.

Hypergarph	|*X*|	|*D*|	rank	mean edge size	median edge size
house-committees	1290	341	82	34.8	40
senate-committees	282	315	31	17.2	19

The proposed heuristic works by removing a set of nodes *S*′ with the size of *k*, having the largest weighted node degree centrality, and then calculating the average weighted node degree centrality. *k* is the same number of nodes removed by the GA in any fitness calculation, and it is the same *k* as the number of critical nodes from the CNDP. This heuristic was chosen since our goal with the GA is similarly to minimize the average weighted node degree centrality. Logically, we could say, that by removing the largest contributors to the average of weighted node degree centrality in the original network, the new average would be strictly better, than removing any other combination of nodes. This comparison proves the contrary since the set of truly critical nodes will further lower the average, and since the GA gives better results, we can consider them nodes that are more similar to critical nodes.

**Figure 4:**
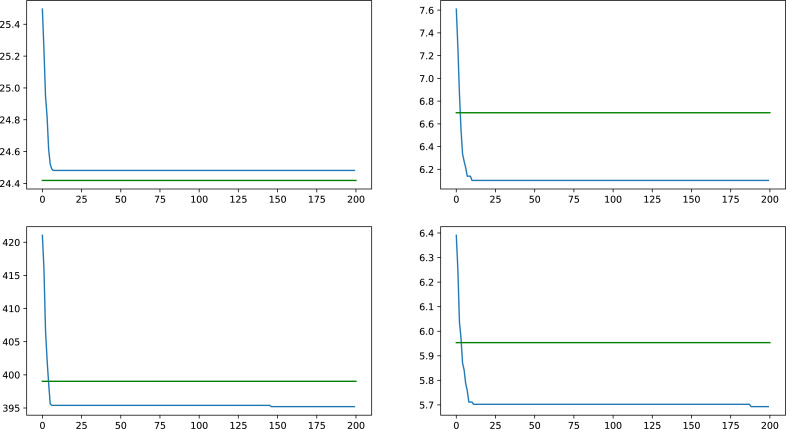
Comparison between heuristic and GA results for a synthetic network on all four tested weight types, values indicate average centrality over time.

Comparisons were made with four synthetic networks, on all weight types, taking results from the average of ten GA runs and comparing them with the result given by the heuristic on the same networks, an overall improvement of 2.57% was observed with differences ranging from similar results, all the way up to an improvement of 14.14%. In Fig. 4 a comparison is presented on a typical synthetic network on the different weight types, with results of the GA presented as the blue lines, while the green lines represent the heuristic result for the same network and weight type.

#### Real world networks

A comparison is given between the best results individuals for each weight type’s run and the result they would have gotten if evaluated by every other weight type. This comparison will be done for both the house and senate networks.

In Tables 3 and 4 an * symbol represents the value obtained by the best run in each category; the best run is chosen instead of an average in order to get a concrete set of nodes that can be tested with the other weight types.

Some interesting results can be read from Tables 3 and 4. Firstly, in general, it can be said that no single weight type produces significantly better results than any other weight type. Secondly, while there is a loose correlation between results, given that the improvement process lowers all types of results, lowering a category doesn’t lower every other category, so there is some categorization to be done. Two categories can be identified: Constant and Frequency-based weights are strongly related according to the results, and the Newman and Network-based approaches are also closely related. The results are around the point where lowering the numbers in one category actually increases the numbers in the other one, which is interesting.

An interpretation of the results is also needed, given that these two networks represent real-world data, more specifically memberships in US house and senate committees. The problem at hand was the detection of critical or important nodes in the networks. This, translated to the committee interpretation, was the problem of finding the ‘most active’ US House members and senators during the period these two data aggregations were made. Ten runs were made with every weight type, this means 40 runs for each network. Next, a few statistics will be given, all conclusions are done on the final results of every run.

**Table 3:** Result comparisons for the house committees network. An asterisk (*) represents the value obtained by the best run in each category.

Original weight type	Constant	Frequency	Newman	Network
Constant	*292.91	293.24	8.32	8.31
Frequency based	290.28	*290.56	8.31	8.30
Newman approach	301.10	301.26	*8.27	8.25
Network approach	299.16	299.39	8.25	*8.24

**Table 4:** Result comparisons for the senate committees network. An asterisk (*) represents the value obtained by the best run in each category.

Original weight type	Constant	Frequency	Newman	Network
Constant	*246.14	252.94	18.43	18.18
Frequency based	245.20	*251.54	18.27	18.07
Newman approach	247.77	254.00	*18.15	17.97
Network approach	256.85	262.23	18.07	*17.94

In the house network, a total of 666 unique nodes out of the 1,290 total nodes were identified at least once in the results as ‘important’. If we give a threshold of appearance of 50% of the runs, then this number lowers to 60 out of 1,290, and if the threshold is raised to 75%, then the result yet again lowers, this time to only two nodes. In contrast, for the senate network, a total of 81 of the 282 nodes appear at least once. With the 50% threshold, this number lowers to 29, and with the 75% threshold, the result is nine nodes.

These statistics are interesting and extremely telling about the nature of both the US house and senate. Senators are lower in number at any given time and are typically re-elected multiple times, giving a more stable population of the US Senate. With members present for many years, they can participate in more committees, and long term, senior members will appear as important, or critical in the data. By contrast, house members are more numerous and experience more movement in their population numbers, so finding a more senior house member, who participated in more committees is hard. An important observation is that the data already provides a filter on both house and senate members since only those who have participated in at least one committee are even represented here.

These statistics could be useful in determining a form of productivity metric among house and senate members, maybe even giving an indication about who were the key players in US politics at any given time, during the historic period processed here. This information can be used in predicting or suggesting re-elections or state-level challenges, or it can be used to determine historical cliques and voting patterns in both chambers.

## Conclusions

A tool for the critical node detection problem in hypergraphs was presented, using weighted node degree centrality measures. Results on synthetic networks proved the validity of this analysis method. Two real-world networks were presented and analyzed using the algorithm provided and some interesting conclusions could be drawn, given the political nature of the presented networks. Analyzing hypergraphs proved to be a subject worthy of investigation, as it can provide a new look into the field of network analysis.

In future work, the introduction of new centrality measures or other types of fitness values should be considered. Possibilities for centrality metrics are large in both number and variety, but each metric has to be investigated in terms of their applicability on hypergraphs. Multiple instances of centrality metrics can be found for example in Rasti & Vogiatzis (2022), where descriptions for multiple centrality metrics are defined such as group degree centrality, group average-closeness centrality, group betweenness centrality, representative degree centrality, clique betweenness centrality, and star closeness centrality. Other types of centralities may need to be analyzed for different types of connections, such as stochastic centralities for random networks or probabilistic connections, such as the row-stochastic centrality presented in Mostagir & Siderius (2021).

Refinement of the algorithm is another possibility, especially creating a variant that can swiftly analyze larger, more complex networks, in order to provide analysis for networks that were previously not analyzed from the critical node detection perspective. One possibility of network applications can be that of protein interaction networks, in Rasti & Vogiatzis (2019) the authors propose, that one of the common representations of protein interactions is protein complex hypergraphs.

## Supplemental Information

10.7717/peerj-cs.1351/supp-1 Supplemental Information 1 Hypergraph of US Senate Committees Each row contains hyperedges representing committees, with each node representing senators and labeled with integers. These were derived from Congressional data compiled by Charles Stewart and Jonathan Woon.

10.7717/peerj-cs.1351/supp-2 Supplemental Information 2 Hypergraph of US House Committees Each row contains hyperedges representing committees, with each node representing house members and labeled with integers. These were derived from Congressional data compiled by Charles Stewart and Jonathan Woon.

10.7717/peerj-cs.1351/supp-3 Supplemental Information 3 Synthetic LFR data 1 Each row contains two numbers. The first represents a hyperedge while the second represents a node. These are synthetic and were constructed using LFR benchmarks. They were used as proof of concept and as parameter-setting tools.

10.7717/peerj-cs.1351/supp-4 Supplemental Information 4 Synthetic LFR data 2 Each row contains two numbers. The first represents a hyperedge while the second represents a node. These are synthetic and were constructed using LFR benchmarks. They were used as proof of concept and as parameter-setting tools.

10.7717/peerj-cs.1351/supp-5 Supplemental Information 5 Synthetic LFR data 3 Each row contains two numbers. The first represents a hyperedge while the second represents a node. These are synthetic and were constructed using LFR benchmarks. They were used as proof of concept and as parameter-setting tools.

10.7717/peerj-cs.1351/supp-6 Supplemental Information 6 Synthetic LFR data 4 Each row contains two numbers. The first represents a hyperedge while the second represents a node. These are synthetic and were constructed using LFR benchmarks. They were used as proof of concept and as parameter-setting tools.

10.7717/peerj-cs.1351/supp-7 Supplemental Information 7 Synthetic LFR data 5 Each row contains two numbers. The first represents a hyperedge while the second represents a node. These are synthetic and were constructed using LFR benchmarks. They were used as proof of concept and as parameter-setting tools.

10.7717/peerj-cs.1351/supp-8 Supplemental Information 8 Synthetic LFR data 6 Each row contains two numbers. The first represents a hyperedge while the second represents a node. These are synthetic and were constructed using LFR benchmarks. They were used as proof of concept and as parameter-setting tools.

10.7717/peerj-cs.1351/supp-9 Supplemental Information 9 The hycrit algorithm proposed for the study This contains multiple functions that correspond to specific parts of the proposed algorithm. It makes use of the HyperNetX python package from the Battelle Memorial Institute.

## Additional Information and Declarations

### Data Availability

The following information was supplied regarding data availability: The raw data and code are available in the Supplemental Files.
